# Evaluation of the Surface Roughness and Color Stability of Two Types of Milled Zirconia Before and After Immersion in Alcoholic Beverages: An In Vitro Study

**DOI:** 10.7759/cureus.64695

**Published:** 2024-07-16

**Authors:** Ray S Arindham, Vidyashree V Nandini, Shiney Boruah, Russia Marimuthu, Surya R, Jailance Lathief

**Affiliations:** 1 Prosthodontics, Sri Ramaswamy Memorial (SRM) Kattankulathur Dental College and Hospital, Chengalpattu, IND

**Keywords:** color stability, surface roughness, alcoholic beverages, zirconia, cad/cam blocks

## Abstract

Objective

This study aims to evaluate the effects of immersion in alcoholic beverages on the surface roughness and color stability of two types of milled zirconia.

Materials and methods

The sample size included 60 cuboid-shaped samples of two types of zirconia (Z1 and Z2), 30 in each group. Zirconia was milled and sintered at 1,500°C for eight hours. The samples were immersed in artificial saliva (control), red wine, and whiskey three times a day over a 30-day period. After each post-immersion cycle, samples were cleaned ultrasonically. Surface roughness and color parameters were measured using an atomic force microscope (AFM) and spectrophotometer before and after immersion. The collected data was organized into tables, and statistical analysis was conducted using the Statistical Package for the Social Sciences (SPSS) version 27 software (IBM SPSS Statistics, Armonk, NY). For surface roughness, a paired t-test was conducted, while for color change, one-way analysis of variance (ANOVA) and Tukey's honestly significant difference (HSD) tests were done.

Results

The mean values of pre- and post-immersion values reveal that whiskey causes the highest difference in surface roughness for Z1 (137.09 nm) and Z2 (86.15 nm) groups, while red wine causes maximum discoloration in both Z1 (2.41) and Z2 (1.94) groups. The paired t-test revealed significant surface roughness changes in Z1 with artificial saliva and red wine, while whiskey (p<0.05), although showing changes, lacked statistical significance (p>0.05). The whiskey group demonstrated a moderate linear association (0.599) between pre- and post-immersion values. For Z2, artificial saliva, red wine, and whiskey (p<0.05) induced statistically significant surface roughness alterations. ANOVA tests indicated significant color changes post-immersion in all three subgroups of Z1 and Z2 (p<0.05 for both). Tukey's HSD test showed significant differences between artificial saliva and red wine (p<0.05), as well as artificial saliva and whiskey (p<0.05) in Z1 and Z2. However, no significant difference was found between red wine and whiskey in both Z1 and Z2 groups (p>0.05).

Conclusion

Whiskey, red wine, and artificial saliva increased zirconia's surface roughness. Alcoholic solutions altered zirconia's colorimetric parameters, with no significant differences among them.

## Introduction

Metal ceramic restorations have been a popular and reliable material in prosthetic dentistry. This is due to their great mechanical properties, reasonably good aesthetic outcomes, and clinically acceptable adaptation over the prepared margins. Over time, porcelain-fused-to-metal crowns and fixed prostheses have become more and more common as the casting procedures and techniques have become simpler without compromising the accuracy of the restorations. Their consistency and predictability of positive clinical results are also backed up by long-term scientific evidence.

While dental tissues are highly translucent, metal ceramic restorations do not have high translucency properties [1]. However, porcelain-fused-to-metal crowns fail to meet these modern aesthetic demands, due to which newer materials had to be researched. Due to its excellent physical, mechanical, and optical properties, zirconia is currently frequently utilized in prosthetic dentistry. Zirconia's qualities and the continued advancement of digital technology have led to its application in the development of implants, short fixed dental prostheses, and individual dental crowns [2]. In fixed prosthodontics, metal-free restorations have become a viable treatment option [1], exhibiting improved cosmetic qualities compared to metal ceramic restorations and good mechanical behavior [3].

The majority of the growth in alcohol consumption per capita in the World Health Organization (WHO) South-East Asia area can be attributed to India, which is still developing quickly. As per the WHO, the annual per capita use of alcohol among those over the age of 15 is 6.2 liters of 100% ethanol every day [4]. According to Lakshmi et al., there is a 42.65% prevalence of alcohol intake and a 38.8% prevalence of problem drinking in Chennai's urban region [4]. Excessive alcohol intake has deleterious effects on teeth and oral tissues. Alcohol, due to its high concentration of organic and inorganic acids, can lead to long-term inflammation of soft tissues and exacerbate the adverse effects of metal restorations such as crowns, bridges, orthodontic devices, and other metal restorations [5].

The erosive potential of alcoholic beverages on dental hard tissues and the clinical efficacy of restorative materials is a concern. A rougher surface promotes faster microbial colonization and biofilm maturation, raising the possibility of dental caries and periodontal disease development, in addition to making the restoration more susceptible to discoloration [6,7]. Given how frequently tooth-colored restorative materials are used, it is critical to identify which ones are prone to color deterioration. Tooth-colored materials must have intrinsic color stability and resistance to surface stains to guarantee excellent aesthetics [7]. Changes in surface texture following various surface treatments may be connected to ceramic staining [8]. Existing literature throws light on alcohol affecting various properties of composites. At the same time, available literature on zirconia is scarce. Hence, this study is conducted with the purpose of evaluating the effect of alcoholic beverages on zirconia. This study began with a null hypothesis that there would be no significant change in surface roughness and color stability in milled zirconia post-immersion in artificial saliva and alcoholic beverages, namely, whiskey and red wine.

## Materials and methods

**Figure 1:**
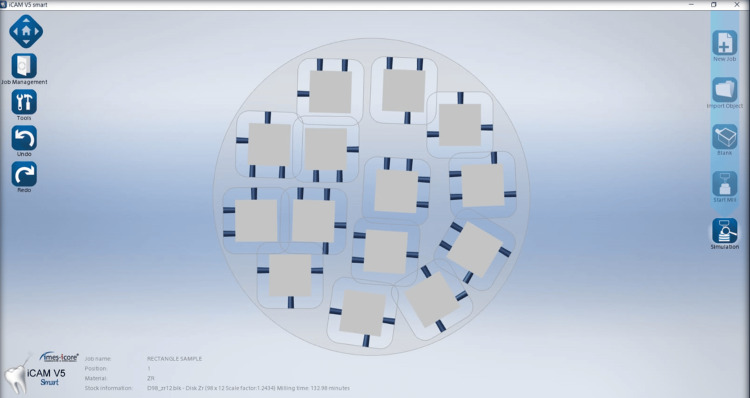
CAD design for milling of specimens CAD: computer-aided designing

**Figure 2:**
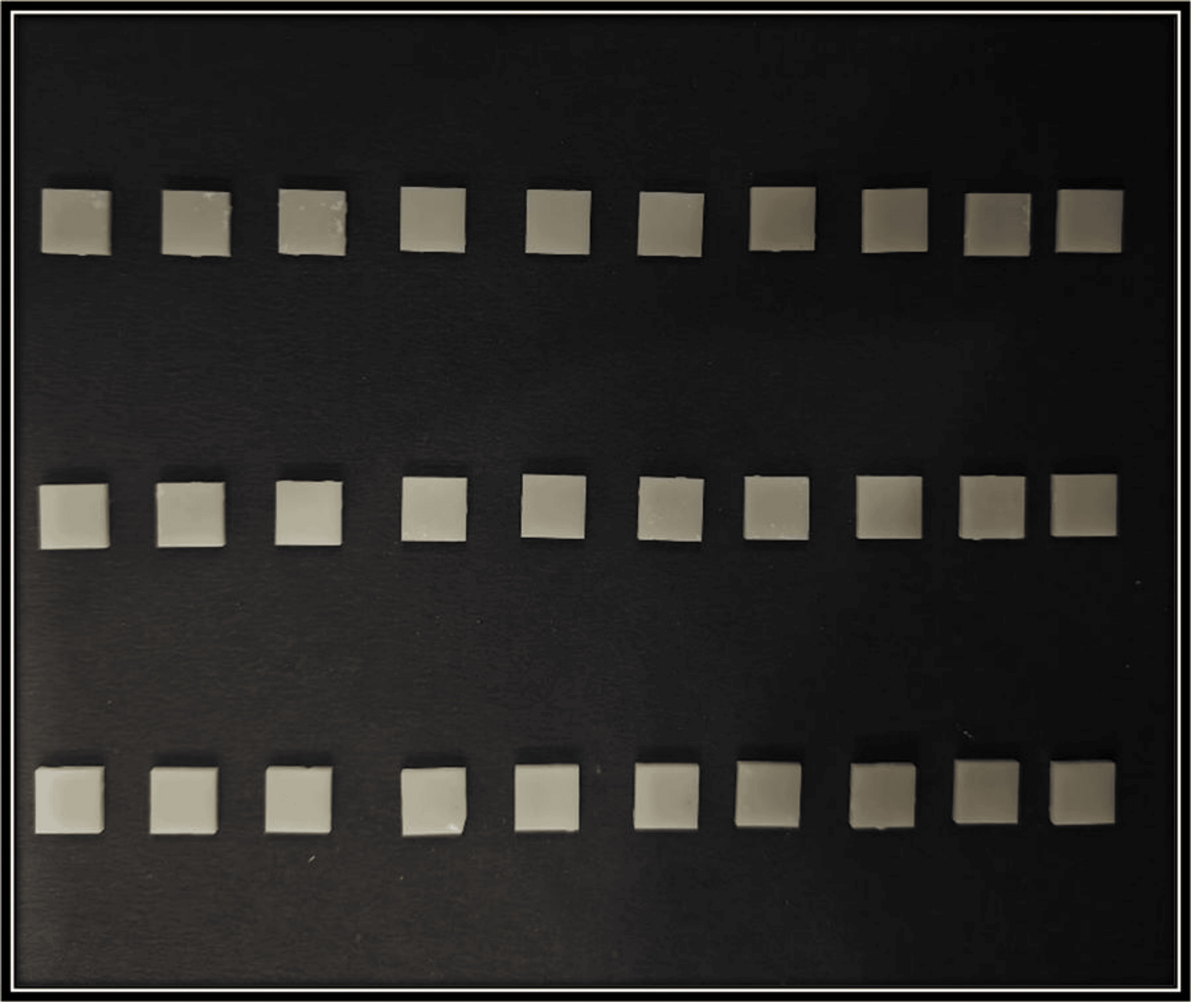
Z1 samples (NexxZr T, Sagemax®, CAD/CAM disc (12 mm), D2 shade, high translucency; Ivoclar Vivadent, Amherst, NY) CAD: computer-aided designing, CAM: computer-aided manufacturing

**Figure 3:**
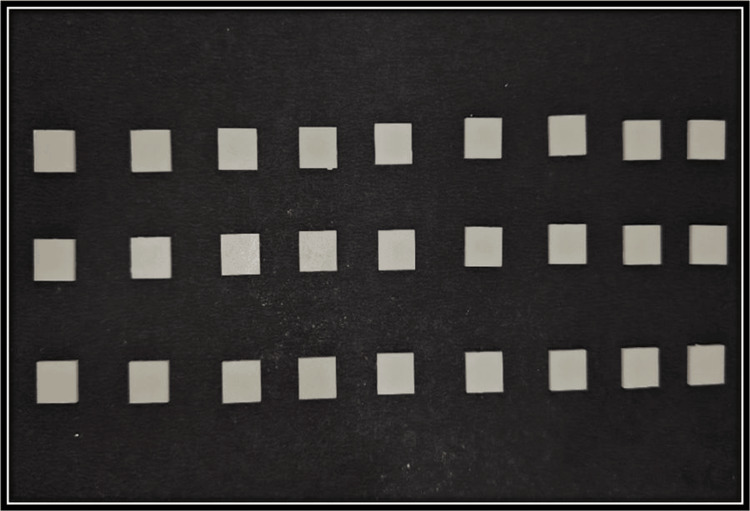
Z2 samples (Cercon®ht, CAD/CAM disc (12 mm), A1 shade, high translucency; Dentsply Sirona, Charlotte, NC) CAD: computer-aided designing, CAM: computer-aided manufacturing

**Figure 4:**
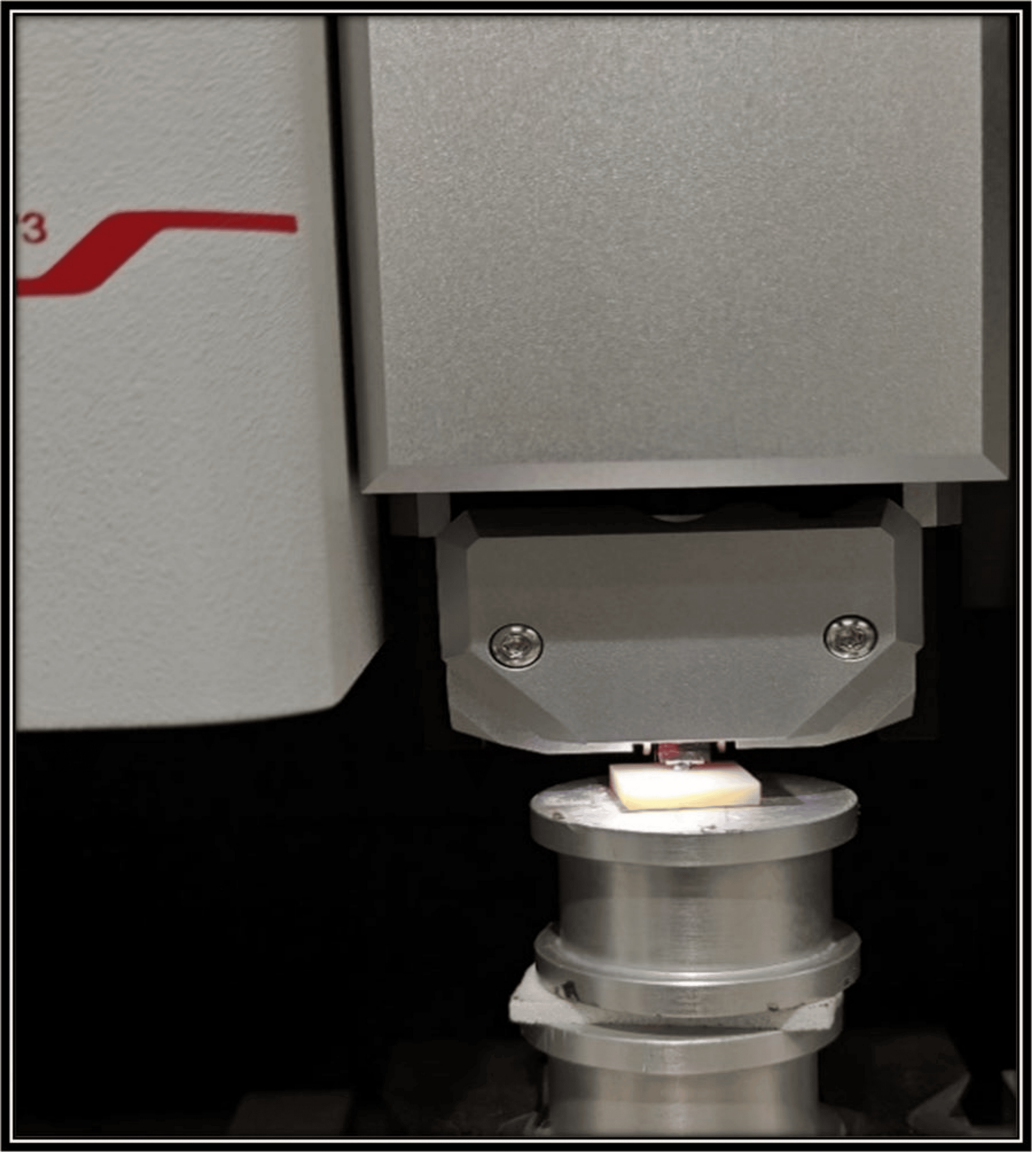
Pre- and post-immersion assessment for surface roughness using an atomic force microscope

This study began with a sample size of 30 in each group. The power of the study is 0.9 (G*Power software version 3.1). This indicates an increased likelihood of correctly identifying a true variation between the two groups if it exists. Firstly, the samples were designed using computer-aided designing (CAD) software (iCAM V5 smart, imes-icore, Eiterfeld, Eastern Hesse, Germany) as depicted in Figure 1. Sixty cuboid-shaped samples of dimension 10 × 10 × 2 mm were fabricated from two types of computer-aided designing/computer-aided manufacturing (CAD/CAM) materials as depicted in Figure 2 and Figure 3 [9]. Dry milling of the samples was done, which were later cleaned ultrasonically and dried. After the completion of the milling of the zirconia samples (NexxZr T, Sagemax®, Ivoclar Vivadent, Amherst, NY, and Cercon®ht, Dentsply Sirona, Charlotte, NC), they were subjected to a sintering process at 1500°C temperature for eight hours, and then, polishing of the samples was done [9]. The samples were cleaned using an ultrasonic bath for 10 minutes. They were carefully stored in borosilicate glass bottles with a capacity of 20 mL. These bottles were labeled based on the groups and subgroups. NexxZr T zirconia was marked as "Z1," while Cercon®ht samples were designated as "Z2." The red wine group was denoted as "R," whiskey as "W," and artificial saliva/control as "C." Each sample was designated a number (1-10 for each subgroup) for ease of identification and increased accuracy of measurements (e.g., Z1C1, Z2R4, Z2W6, and Z1C9). Sixty samples were taken and divided into two groups of 30 each and three subgroups for each group having 10 samples in each subgroup for the study. Surface roughness (Figure 4) and color stability of samples were checked prior to and post-immersion in different solutions. Surface roughness was measured using an atomic force microscope (Anton Paar, Graz, Austria), and color stability was measured using a color reflectance spectrophotometer (CM5 Konica Minolta, Tokyo, Japan). For surface roughness, Ra values were checked, while for color stability, ΔE values were measured using the CIE L*a* b system.

Samples were transferred to the labeled bottles. Solutions (10 mL) were added to the borosilicate bottles based on the group using a borosilicate measuring cylinder. The milled specimen was submersed in the respective alcoholic beverages (red wine in the "R"-labeled bottles and whiskey in "W"-labeled bottles) for 15 minutes. After 15 minutes, the alcoholic solutions were discarded, samples were cleaned in an ultrasonic cleaner for 10 minutes, and then, they were dried for 10 minutes. Following this, the samples were placed in artificial saliva to simulate oral clinical conditions. This immersion procedure was done three times a day at 8:00 am, 2:00 pm, and 9:00 pm. This procedure was repeated over a span of 30 days [6]. After the entire immersion procedure, the samples were cleaned and dried.

Then, surface roughness and color stability parameter measurements were carried out post-immersion after 30 days. The results were subjected to statistical analysis using the Statistical Package for the Social Sciences (SPSS) version 27 software (IBM SPSS Statistics, Armonk, NY). Paired t-tests collated the means prior to and post-immersion for surface roughness. For color stability, analysis of variance (ANOVA) and Tukey's honestly significant difference (HSD) tests were done.

## Results

The mean surface roughness values pre- and post-immersion in artificial saliva, red wine, and whiskey for the Z1 group were 84.97, 92.97, and 102.42 nm, and 100.79, 116.74, and 137.09 nm, respectively. The mean surface roughness values pre- and post-immersion in artificial saliva, red wine, and whiskey for the Z2 group were 70.54, 65.34, and 60.66 nm, and 77.26, 89.16, and 86.15 nm, respectively.

The mean color variation values post-immersion in artificial saliva, red wine, and whiskey for the Z1 group were 1.25, 2.41, and 1.93, respectively, while the mean color variation values post-immersion in artificial saliva, red wine, and whiskey for the Z2 group were 1.01, 1.94, and 1.61, respectively.

Z1 (Sagemax®) and Z2 (Cercon®ht) samples have shown an increase in surface roughness post-immersion in artificial saliva, red wine, and whiskey. Paired sample t-test reveals significant differences (p<0.05) (Table 1). It was observed that whiskey induced the most significant alteration in surface roughness, trailed by red wine, with artificial saliva showing the least impact in both Z1 and Z2 groups.

**Table 1:** Paired sample t-test for the comparison of two different zirconia groups for surface roughness before and after immersion Z1: Sagemax®, NexxZr T, translucent dental zirconia, Z2: Dentsply Sirona, Cercon®ht, true color technology, C: control, R: red wine, W: whiskey

Pair	Pre- and post-immersion difference (surface roughness)	Mean	Standard deviation	Standard error of the mean	95% confidence interval of the difference		t	df	Sig(2-tailed)
					Lower	Upper			
Pair 1	Z1C pre-immersion (nm) - Z1C post-immersion (nm)	-15.81970	8.95946	2.83323	-22.22891	-9.41049	-5.584	9	0.000
Pair 2	Z1R pre-immersion (nm) - Z1R post-immersion (nm)	-23.76700	17.07438	5.39939	-35.98128	-11.55272	-4.402	9	0.002
Pair 3	Z1W pre-immersion (nm) - Z1W post-immersion (nm)	-34.65910	22.52734	7.12377	-50.77419	-18.54401	-4.865	9	0.001
Pair 4	Z2C pre-immersion (nm) - Z2C post-immersion (nm)	-6.71730	2.99701	0.94774	-8.86123	-4.57337	-7.088	9	0.000
Pair 5	Z2R pre-immersion (nm) - Z2R post-immersion (nm)	-23.82040	11.68056	3.69372	-32.17617	-15.46463	-6.449	9	0.000
Pair 6	Z2W pre-immersion (nm) - Z2W post-immersion (nm)	-25.48700	8.06179	2.54936	-31.25406	-19.71994	-9.997	9	0.000

Z1 (Sagemax®) and Z2 (Cercon®ht) samples exhibited notable color alteration after immersion in artificial saliva, red wine, and whiskey. ANOVA test indicates significant differences (p<0.05). Post hoc Tukey's HSD test indicates that both red wine and whiskey elicit significant color change in both the Z1 and Z2 samples when compared to artificial saliva as the p-value is lower than 0.05. However, when the color change caused by whiskey and red wine in both Z1 and Z2 samples were compared to each other, it was found out to be non-significant as p-values are greater than 0.05 (Table 2 and Table 3).

**Table 2:** Tukey's HSD for Z1 color change after immersion *Significant Z1: Sagemax® NexxZr T, translucent dental zirconia, HSD: honestly significant difference, C: control, R: red wine, W: whiskey

Group Z1 (I)	Group Z1 (J)	Mean difference (I-J)	Standard error	Significance	95% confidence interval	
					Lower	Upper
Z1C	Z1R	-1.16200^*^	0.27590	0.001*	-1.8461	-0.4779
	Z1W	-0.68700^*^	0.27590	0.049*	-1.3711	-0.0029
Z1R	Z1C	1.16200^*^	0.27590	0.001*	0.4779	1.8461
	Z1W	0.47500	0.27590	0.216	-0.2091	1.1591
Z1W	Z1C	0.68700^*^	0.27590	0.049*	0.0029	1.3711
	Z1R	-0.47500	0.27590	0.216	-1.1591	0.2091

**Table 3:** Tukey's HSD for Z2 color change after immersion *Significant Z2: Dentsply Sirona Cercon®ht, true color technology, C: control, R: red wine, W: whiskey

Group Z2 (I)	Group Z2 (J)	Mean difference (I-J)	Standard error	Significance	95% confidence interval	
					Lower	Upper
Z2C	Z2R	-0.92900^*^	0.22978	0.001*	-1.4987	-0.3593
	Z2W	-0.60300^*^	0.22978	0.036*	-1.1727	-0.0333
Z2R	Z2C	0.92900^*^	0.22978	0.001*	0.3593	1.4987
	Z2W	0.32600	0.22978	0.346	-0.2437	0.8957
Z2W	Z2C	0.60300^*^	0.22978	0.036*	0.0333	1.1727
	Z2R	-0.32600	0.22978	0.346	-0.8957	0.2437

## Discussion

Utilizing CAD/CAM technology, crafting a zirconia prosthesis is now a quick and efficient process, allowing for high-quality work to be accomplished in a relatively short period. Around the world, a wide variety of alcoholic beverages are enjoyed. Beer, wine, whiskey, rum, vodka, gin, and brandy are the most widely consumed alcoholic beverages. Locally produced drinks such as arrack and toddy are also popular. When someone has a problematic drinking pattern that increases his likelihood of experiencing unfavorable health events, alcohol use becomes an issue [4]. Finally, alcohol control initiatives are implemented in a fragmented, non-consensual manner, affected by social factors, and motivated by limited economic rather than health concerns [10].

Khairnar et al. [5] reported that alcoholics typically have a higher rate of tooth decay, which results in either tooth extraction (missing teeth) or tooth repair (fillings). To rehabilitate such cases, crowns and bridges can be considered as good treatment options.

Alcoholic beverages have proven to be one of the many extrinsic factors leading to dental erosion. Rougher surface promotes faster microbial colonization and biofilm maturation, raising the risk of dental caries and periodontal disease development as well as the restoration's sensitivity to staining [6]. Pigments in food and drinks produce deposition and adsorption that results in the color change of dental restorations. Discoloration typically gets worse with time and varies in intensity based on the type of restorative material used and the characteristics of materials containing the pigments [11]. Da Silva et al. [6] stated that in the presence of organic acids, degradation is notably more pronounced by whiskey (40% alcohol by volume (ABV), pH 3.76), which emerged as the most aggressive beverage, resulting in significant surface degradation of composites. Because of the red-colored pigments in red wine, it can have an impact on color stability [12]. Hence, this study aimed to find the effects of different types of alcoholic beverages on the surface roughness and color of two brands of milled zirconia samples.

Current study results reveal that for both the Z1 group (NexxZr T, Sagemax®, Ivoclar Vivadent, Amherst, NY) and Z2 group (Cercon®ht, Dentsply Sirona, Charlotte, NC), artificial saliva, red wine, and whiskey had caused statistically significant changes in surface roughness post-immersion. The results suggest that whiskey, red wine, and even artificial saliva caused an increase in surface roughness after immersion. Da Silva et al. [6] have suggested that pH plays a crucial role in determining the erosive potential of a solution. Whiskey has been known to cause an increase in surface roughness in dental composites. In the presence of organic acids, it is considered to be extremely aggressive. It is also suggested that low pH and high alcohol content can be contributing factors to its aggressive nature.

Tukey's HSD test revealed that for the Z1 and Z2 groups, there was a statistically significant difference between artificial saliva and red wine, as well as artificial saliva and whiskey, but there was no statistically significant difference between red wine and whiskey. The study results suggest that alcoholic beverages such as red wine and whiskey caused significant color changes in both zirconia groups as compared to artificial saliva. The amount of color change caused by the different alcoholic beverages studied was found to be similar to each other. When the mean values were compared, it was observed that red wine caused more colorimetric changes as compared to whiskey in Z1 and Z2 samples. However, the color changes, when compared with each other for alcoholic beverages, were statistically not significant. Hence, any alcoholic solution could cause a change in colorimetric parameters in zirconia. These findings contradict the null hypothesis, as it can be concluded that alcoholic beverages indeed induce alterations in the surface roughness and color parameters of zirconia.

Alcoholic solutions are known to damage surfaces due to their erosive potential; it has been suggested that this also leads to discoloration. In red wine, there is a red dye called anthocyanin, which is known to cause extrinsic stains. Due to its low pH, red wine is also known to cause damage to surface integrity [13]. Due to the damage caused by it on the surface and the presence of anthocyanin, it can be suggested to be the reason behind its increased staining potential. Since both whiskey and red wine are alcoholic beverages having low pH, the amount of staining caused by them when compared to each other is not statistically significant. Da Silva et al. [6] have reported the pH of artificial saliva and whiskey as 7.1 and 3.76, respectively. Saba et al. [13] have reported the pH of red wine to be in a range of 3.5-4. They also stated that materials submerged in solutions with pH levels ranging from 4 to 6 exhibited higher sorption values. From the reported data, we can say that whiskey and red wine have similar pH and have lower pH than artificial saliva. This explains how both red wine and whiskey have similar effects on zirconia and both of them cause greater change in surface and color parameters as compared to artificial saliva. In addition to this, the dye in red wine is polar in nature and water soluble [13]. Hence, while cleaning the samples post-immersion, the dye must have cleared off, leading to the color change comparison between wine and whiskey to be not statistically significant. So, the factor causing the change in colorimetric parameters can be majorly attributed to the surface damage caused by these beverages, increasing the susceptibility of zirconia samples to change in colorimetric parameters. Nascimento Oliveira et al. [12] have suggested that the staining of zirconia can be dependent on various factors, such as pH, immersion protocol, and type of liquid. The findings in our current study support the results reported by Barutçugil et al. [14]. Their observations revealed that the zirconia blocks subjected to red wine and coffee immersion for one month displayed a higher degree of discoloration compared to those immersed in water.

The study has been performed to simulate the natural conditions as much as possible, yet this study has a few limitations. In future studies, the CIEDE2000 formula could be employed to examine color differences, as recent studies suggest its superiority in capturing subtle color variations according to human perception [15]. The CIEDE2000 formula offers an enhanced method for calculating color differences in industrial applications [16]. Although the current study has given us promising results, the thermocycling of samples could provide more accurate results and can be incorporated into the methodology of future research. The effects can be further evaluated in an in vivo setting as this study has not taken into consideration factors such as the usage of toothbrushes and other oral hygiene aids.

Consumption of alcoholic beverages such as red wine and whiskey causes changes on the surface of zirconia and also causes changes in color. The deleterious effects of alcohol consumption on zirconia restorations must be informed before treatment planning and can be included as a postoperative instruction.

## Conclusions

Within the limitations of this study, the conclusions drawn were that zirconia had shown an increase in surface roughness post-immersion in artificial saliva/red wine/whiskey. Whiskey induced the most significant alteration in surface roughness, trailed by red wine, with artificial saliva showing the least impact in both types of zirconia.

Zirconia also exhibited notable color alterations after immersion in artificial saliva/red wine/whiskey. Red wine prompts the most pronounced color alteration, followed by whiskey, while artificial saliva exhibited the least impact on zirconia. Alcoholic beverages exhibited significant color change compared to artificial saliva. However, the color changes produced by the different alcoholic beverages were comparable and not contrasting.
